# Evaluating the Toxicity of Chemical Mixtures

**DOI:** 10.1289/ehp.112-a729

**Published:** 2004-09

**Authors:** Gerald A. LeBlanc, Allen W. Olmstead

**Affiliations:** Department of Environmental and Molecular Toxicology, North Carolina State University, Raleigh, North Carolina, E-mail: ga_leblanc@ncsu.edu

Tinwell and Ashby (2004) provided a detailed evaluation of the joint action of a mixture of estrogenic chemicals using the immature rat uterotrophic assay. The researchers demonstrated that a mixture of estrogenic chemicals in which each individual chemical was present in the mixture at levels approximating the no observed effect level (NOEL) elicited a measurable response. This work advances our understanding of the toxicity of endocrine-active substances, and Tinwell and Ashby are to be commended for providing detailed results of their experiments suitable for evaluation by others.

The analysis of the data, however, stopped short of providing insights into the joint action of mixtures of endocrine disruptors. Tinwell and Ashby (2004) proposed three avenues for the analysis of the joint action of chemicals. The first, a simple addition-of-effects approach, is overly simplistic and unrealistic, as demonstrated by the authors. The second, graphic isobole analysis, was rejected by the authors for any mixture in excess of three chemicals. We concur that isobole analysis poses limitations for more complex mixtures of chemicals. The third, concentration addition, was deemed impractical by Tinwell and Ashby due to the requirement of detailed characterization of the concentration–response relationship of each chemical within the mixture. We agree that analysis of mixtures toxicity using concentration addition requires an understanding of the toxicity of the individual constituents within a mixture. However, we disagree that such a data requirement should discourage efforts to model and predict toxicity of chemical mixtures using this approach. Results reported by Tinwell and Ashby (2004), along with published data cited by the authors, provided sufficient information on the toxicity of the individual chemicals for us to accurately model the joint action of the mixture based upon concentration addition.

The authors’ recommendation that toxicity of chemical mixtures be directly assessed on a case-by-case basis (Tinwell and Ashby 2004) would provide a Band-Aid but not a cure to the dilemma of characterizing the hazards of chemical mixtures. Chemical mixtures are ever varying with respect to constituents and to concentrations of those constituents. Granted, the individual toxicity of many, if not most, chemicals has not been adequately evaluated to provide the concentration–response information required for the joint evaluation of toxicity. Rather than avoid such endeavors, the scientific community should mobilize to generate such data; the data should be made available in the public domain; and, alternative approaches (i.e., *in vitro* analyses of ligand–receptor interactions) should be explored as means to rapidly generate surrogate data for use in mixtures toxicity assessments. Thanks to the efforts of investigators such as Tinwell and Ashby, who are generous with the data they have generated, a growing database exists for estrogenic chemicals. Hopefully, key agencies (i.e., the National Institute of Environmental Health Sciences, the U.S. Environmental Protection Agency) will take the initiative to generate public-domain databases on chemicals harboring other mechanisms of toxicity. With such data resources, we may someday have the ability to routinely model the toxicity of chemical mixtures.

## References

[b1-ehp0112-a0729a] Tinwell H, Ashby J (2004). Sensitivity of the immature rat uterotrophic assay to mixtures of estrogens. Environ Health Perspect.

